# Phospholipids: Pulling Back the Actin Curtain for Granule Delivery to the Immune Synapse

**DOI:** 10.3389/fimmu.2019.00700

**Published:** 2019-04-11

**Authors:** Christian M. Gawden-Bone, Gillian M. Griffiths

**Affiliations:** Cambridge Institute of Medical Research, University of Cambridge, Cambridge, United Kingdom

**Keywords:** T cells, cytotoxic T lymphocytes (CTLs), phospholipids, phosphatidylinositol 4,5-bisphosphate (PI(4,5)P2), diacylglycerol (DAG), phosphatidylinositol 3,4,5-trisphosphate (PI(3,4,5)P3)

## Abstract

Phosphoinositides, together with the phospholipids phosphatidylserine and phosphatidic acid, are important components of the plasma membrane acting as second messengers that, with diacylglycerol, regulate a diverse range of signaling events converting extracellular changes into cellular responses. Local changes in their distribution and membrane charge on the inner leaflet of the plasma membrane play important roles in immune cell function. Here we discuss their distribution and regulators highlighting the importance of membrane changes across the immune synapse on the cytoskeleton and the impact on the function of cytotoxic T lymphocytes.

## Cytotoxic T Cells, the Immune Synapse and Phospholipids

Cytotoxic T lymphocytes (CTLs) are important for the clearance of cancerous and virally infected cells. Their task is more difficult as cancers and virally infected organs present as a mosaic of diseased and healthy cells within the tissue. CTLs specifically target infected or cancerous cells using the T cell receptor (TCR) recognizing peptide-loaded, major histocompatibility complex class one (pMHC I) on the surface of an antigen presenting cell [APC; reviewed in (1)]. TCR activation triggers a cascade of signaling events that results in the formation of a characteristic cell-to-cell contact between APCs and the CTLs referred to as the immune synapse [reviewed in (2)]. In CTL TCR clusters at the center of the synapse and lytic granule secretion occurs at a specialized secretory domain, next to the site of signaling (3, 4).

Actin depletion is an essential event that regulates lytic granule secretion at the synapse of both CTLs and natural killer cells (5–8). Although phospholipids and phosphoinositides have been well-characterized as regulators of actin reorganization during phagocytosis and macropinocytosis (9), their role in actin reorganization at the synapse has only recently emerged with more accurate lipid probes allowing visualization of their distribution and regulation across the synapse (10).

**Figure 1:**
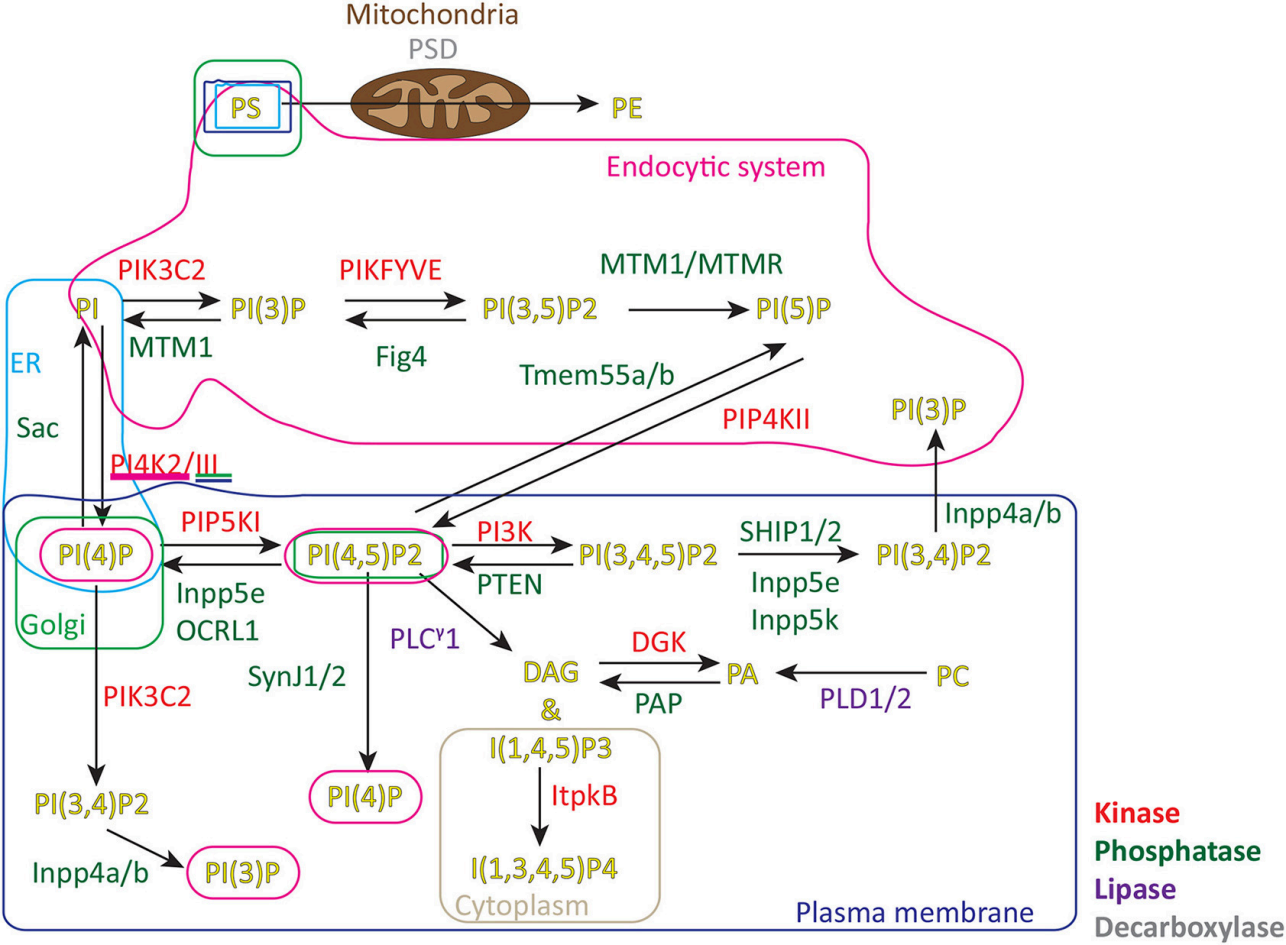
Metabolic pathways and location of signaling lipids in cells. PS, phosphatidylserine; PE, phosphatidylethanolamine; PA, phosphatidic acid; PC, phosphatidylcholine; PI, phosphatidylinositol; PI(3)P, phosphatidylinositol 3-phosphate; PI(4)P, phosphatidylinositol 4-phosphate; PI(5)P, phosphatidylinositol 5-phosphate; PI(4,5)P2, phosphatidylinositol 4,5-bisphosphate; PI(3,4)P2, phosphatidylinositol 3,4-bisphosphate; PI(4,5)P2, phosphatidylinositol 3,5-bisphosphate; PI(3,4,5)P3, phosphatidylinositol 3,4,5-trisphosphate; DAG, diacylglycerol; I(1,4,5)P3, inositol 1,4,5-trisphosphate; I(1,3,4,5)P4, inositol 1,3,4,5-tetrakisphosphate; PSD, phosphatidylserine decarboxylase; PIK3C2, Phosphatidylinositol 4-phosphate 3-kinase C2 domain-containing subunit α/β/γ; PIKFYVE, Phosphatidylinositol 3-phosphate 5-kinase/FYVE finger-containing phosphoinositide kinase; MTM1/MTMR, Myotubularin/Myotubularin related 1-14; Fig4 Polyphosphoinositide phosphatase; Tmem55b/a, Type 2 phosphatidylinositol 4,5-bisphosphate 4-phosphatase/Type 1 phosphatidylinositol 4,5-bisphosphate 4-phosphatase; PIP4KII Phosphatidylinositol 5-phosphate 4-kinase type-2α/β/γ; PI4K2/III, Phosphatidylinositol 4-kinase type 2α/β/Phosphatidylinositol 4-kinaseα/β; PIP5K, Phosphatidylinositol 4-phosphate 5-kinase α/β/γ; PI3K, Phosphatidylinositol 4,5-bisphosphate 3-kinaseα/β/γ/δ; SHIP1/2, SH2 domain-containing inositol 5′-phosphatase 1/SH2 domain-containing inositol 5′-phosphatase 2; Inpp5e, Inositol polyphosphate 5-phosphatase E; OCRL1, Inositol polyphosphate 5-phosphatase; SynJ1/2, Synaptic inositol 1,4,5-trisphosphate 5-phosphatase1/2; PTEN, Phosphatidylinositol 3,4,5-trisphosphate 3-phosphatase and dual-specificity protein phosphatase; Inpp5k, Inositol polyphosphate 5-phosphatase K; PLCγ1, phospholipase C γ1; DGK, diacylglycerol kinaseα/ζ PAP PLD1/2 phospholipase D1/2; Inpp4a/b, Type I inositol 3,4-bisphosphate 4-phosphatase/Type II inositol 3,4-bisphosphate 4-phosphatase; ER, endoplasmic reticulum.

**Figure 2:**
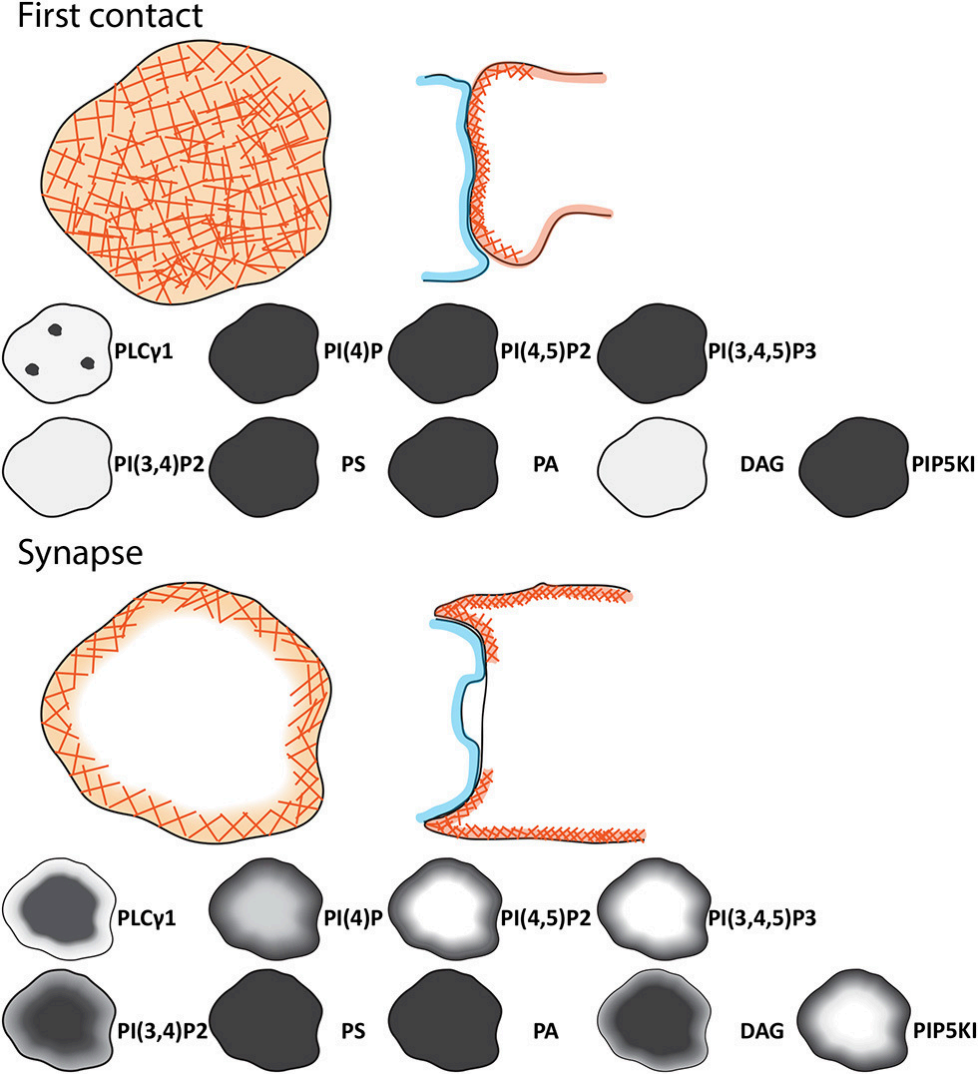
Schematic of the signaling events during initial contact and across the immune synapse of CTL. Cartoons showing the changes in distributions of phosphoinositides, phospholipids, DAG, and regulators between the initial contact between CTL (light orange) and APC (light blue) and the synapse formed. Lower panel provide details on the lipid content of the membrane at the observed point. As the synapse matures the cSMAC (dark red), pSMAC (green), and dSMAC (light orange) form together with the secretory domain (blue). F-actin (orange hashes) PI(4)P, PI(4,5)P2, PIP5K, and PI(3,4,5)P3 are depleted and PLCγ1, PI(3,4)P2 and DAG accumulate across the center of the synapse as indicated by the intensity on the gray scale.

Figure 1 shows the metabolic pathways involved in lipid signaling events within the plasma membrane and endocytic system. Upon initial contact between the CTLs and APCs, F-actin initially accumulates across the forming interface before rapidly depleting as TCR activation triggers changes in the lipid composition across the immune synapse (7, 11). These changes are initiated by TCR activation of the LAT signalosome with Slp76, Gads and phospholipase C gamma 1 (PLCγ1) which rapidly accumulates in clusters as the synapse forms, recruiting other effector proteins that together potentiate signaling (12, 13). DAG, the product of PLCγ1 mediated cleavage of phosphatidylinositol 4,5-bisphosphate (PI(4,5)P2), accumulates as PI(4,5)P2 diminishes across the synapse. Together with other changes an area of membrane specialization is formed (Figure 2).

## Lipid Regulation Generates Membrane Specialization

Low levels of phosphoinositides in membranes allow for exquisite regulation of signaling, with small changes modulating recruitment of signaling proteins. In CTLs the key event in response to changes initiated by TCR activation of PLCγ1 is the loss of PI(4,5)P2 across the immune synapse, which results in a loss of cortical actin across the membrane, allowing granule secretion to occur (10). Equally important to events driving the loss of actin across the synapse are the mechanisms that prevent PI(4,5)P2 being rapidly replenished by the PIP5 kinases (PIP5K). Although PIP5K family members are recruited to the immune synapse in Jurkat cells, these studies did not examine dynamic temporal events (14, 15). When PIP5K dynamics were examined it was found that these kinases are also depleted across the synapse upon TCR activation, and the ability to replenish PI(4,5)P2 via this pathway is also lost (10). Exactly how this coordinated loss of PI(4,5)P2 and the kinase that usually maintains PI(4,5)P2 levels in the plasma membrane occurs in response to TCR activation also emerged from these studies. Several different mechanisms have been shown to control PIP5K membrane association in different cell types, including recruitment via Rac1, ARF6, AP-2, beta-arrestin, talin, and PI(4)P (16–20). However, in macrophages the mechanism for PIP5K membrane association occurs via an electrostatic switch, by virtue of a series of polybasic amino acids that project from a conserved alpha-helix and interact with negatively charged PI(4,5)P2 in the membrane (21, 22). This electrostatic mechanism also functions in T cells and provides a very elegant way of coupling TCR activation with membrane changes that control secretion. Upon TCR activation the loss of PI(4,5)P2 by PLCγ1 also causes a loss of negative charge as DAG replaces PI(4,5)P2. This rapid loss of negative charge causes PIP5K to dissociate from the plasma membrane across the synapse so that PI(4,5)P2 can no longer be replenished via this route. This rapid loss of PI(4,5)P2 drives a rapid loss of actin favoring granule secretion (10). In this way, a small shift in the balance of phosphoinositides is able to control CTL killing.

One challenge to maintaining lipid specialization is that most lipids are highly diffusible within the plasma membrane. DAG has been reported to be a highly diffusible lipid when tracked with the PKC-theta C1 domain probe in B cells (23); nevertheless a DAG gradient is maintained at the synapse membrane by the interplay of lipases and kinases (24). Using diacylglycerol kinase (DGK) α or ζ null mice and TIRFM on activating membranes this study shows that CD8 and CD4 T cells deficient in DGKα have a centrosome polarization defect. This was due to the diffuse distribution of DAG across the synaptic membrane suggesting that the restriction of DAG in the cSMAC is an important feature for centrosome recruitment to the membrane and that DGKα acts to ensure the focus of DAG in the membrane. Importantly, DGKα localized to the dSMAC suggesting it fences in the DAG to the central synapse (24). Any DAG that moves toward the periphery at the concentration of active DGKα is metabolized to PA, which may also constrain RasGrp signaling to the central synapse (25, 26). This limits DAG to a relatively tight area of the membrane and this in turn maintains the polarizing signal (24). More importantly, DAG would create a spatially regulated docking site for centrosome delivery to the synapse. DGKζ deletion had no effect on the spatial regulation of DAG at the synapse and did not prevent centrosome polarization (24). Although, the role of DGKζ is less clear at the synapse where it is implicated in the regulation of signal strength at the synapse through its control of the Ras and Erk pathways (27).

Intriguingly many other proteins have specific domains or positively charged outer surfaces that allow sensing of charge changes at the plasma membrane [reviewed in (21),(28–32)]. A number of synthetic probes exist to detect charge in the membrane, based on arginine and lysine rich amino acid chains, with a lipidation motif at their C-terminus. These include the Kras+8 probe, R-pre probe, and the MCS+ probe based on R-pre [described in detail in (32–34)]. The drop in charge in both the CTL synapse and in Jurkat cells has been detected with these probes (10, 33), suggesting this is a general mechanism for modulating the immune synapse membrane.

## PI(4,5)P2, PIP5K, and PLCγ1 Regulate the Actin Cytoskeleton in the Synapse

F-actin dynamics at the synapse are important for TCR signaling and delivery of granules to the synapse membrane (7, 35, 36). Several lines of evidence support a link between the loss of PI(4,5)P2 and the loss of actin across the synapse. First, the loss of PI(4,5)P2 correlates both spatially and temporally with the loss of actin [(7); Figure 2]. Second, the phospholipase C inhibitor, U73122, which inhibits PLCγ1, blocks actin loss from the central synapse region. Thirdly, blocking the depletion of PIP5K proteins from the synapse by tagging PIP5Kβ with the palmitoylation domain of Lyn protein, blocks actin depletion across the synapse.

The role of PI(4,5)P2 in recruiting actin to the cortical membrane is mediated by a number of actin-recruiting proteins that bind directly to PI(4,5)P2. These include the Ezrin/Radaxin/Moesin (ERM) proteins which interact with PI(4,5)P2 via their FERM domains (37). Phosphorylation of ERM proteins stabilizes their open conformation and allows for bridging of the actin cytoskeleton and PI(4,5)P2 (38, 39). PIP5Ks have also been shown to recruit ERM to the membrane of primary 5C.C7 TCR T cells by production of PI(4,5)P2 (40). Interestingly, ERM proteins deplete across the CD4 T cell synapse after initial contact between T cell and APC (41). Furthermore, cleavage of PI(4,5)P2 by PLCγ1 triggers depletion of Ezrin from the membrane in CTLs (39).

Wiskott-Aldrich syndrome protein (WASp) also plays a role in actin recruitment, interacting directly with PI(4,5)P2 via a polybasic region. Interestingly, WASp has a direct role in TCR activation and regulation of PLCγ1 activity in the CTL membrane. WASp recruitment to TCR complexes is thought to be required for efficient activation of TCR and the subsequent recruitment of PLCγ1, PI(4,5)P2 metabolism and calcium signaling; all of which were defective in WASp null T cells (36). This suggests that TCR activation via WASp occurs early in signaling when the membrane has abundant levels of PI(4,5)P2, or occurs later at the periphery of the maturing synapse where PI(4,5)P2 is also abundant. Alternatively, low levels of PI(4,5)P2 present in the central synapse might be sufficient to recruit WASp, or perhaps WASp might be recruited via PI(3,4)P2, which is a mirror image of the PI(4,5)P2 and is abundant at the central synapse membrane.

## PI(3,4,5)P3 Distributions Lead to Forces Across the Synapse

Most research to date has focused on understanding the interplay of signaling proteins on the CTL side of the immune synapse. However, this is only half the story in the interaction between T cell and target. The membrane tension generated on the target side of the synapse is an important regulator of perforin-mediated killing. Phosphoinositides, actin and integrins are all thought to mediate forces sensed by the target. The phosphoinositide PI(3,4,5)P3 controls actin movement in the periphery through Rac1 activity, a potent regulator of actin cytoskeletal dynamics. These actin mediated forces are focused in the dSMAC where engulfment and cell motility protein 1 (ELMO) is recruited to the plasma membrane via PI(3,4,5)P3. Here it interacts with dedicator of cytokinesis protein 2 (DOCK2) activating Rac1 to initiate WAVE driven actin dynamics (42).

More recently a role for PI(3,4,5)P3 in force generation at the synapse was demonstrated using deflection of micropillars to measure forces. Consistent with the earlier findings, loss of DOCK2 resulted in a loss of force across the synapse (43). This elegant system was also able to reveal force direction and showed that early forces pushed in to the periphery in a process termed mechanopotentiation. These results suggested that these forces, which were myosin II dependent and were increased when PTEN was silenced, increased membrane tension across the APC membrane, potentiating perforin-mediated killing (35, 43).

Integrin interactions, supported by the local lipid microenvironment, also play an important role in generating actin-driven forces across the synapse. Integrin activators kindlin-3 and talin, which stabilize the extended conformation of the integrin in to the extracellular milieu, depend on the presence of PI(3,4,5)P3 and PI(4,5)P2 (44, 45). Kindlin-3 may be dispensable for synapse formation and integrin activation in T cells (46–48), whereas talin and vinculin's interaction with integrins drives TCR responses in CD4 T cells (49). PI(3,4,5)P3 is thought to drive actin flow at the periphery of the synapse which in turn regulates integrin and WAVE activation (43). Integrins are drawn in the direction of the actin flow in CTLs, with continued ruffling between CTLs and APC generating tension (50). The actin cytoskeleton in the dendritic cell also supports T cell interaction through the stabilization of ICAM-1contributing to the forces generated (51). Although the direction of actin flow in the synapse is controversial, with TIRF imaging interpreted as showing a centripetal inward flow of actin to the dSMAC (52), while lattice light sheet showed a rearward flow of peripheral lamellipodial actin toward the uropod of CTL (7), the direction of actin flow in the synapse will have an important role in integrin activation and forces generated at the synapse.

TCR activation in the periphery also drives inside out integrin activation and tension creation, as CasL, a phosphoprotein with multiple kinase docking sites is recruited to the peripheral TCR complexes and support larger TCR clusters when present. CasL recruitment led to integrin activation at the immune synapse (53), further building on the hypothesis that continued actin dynamics, TCR activation and integrin activation support each other in synapse formation. Activation of integrin leukocyte function-associated molecule 1 (LFA-1; CD11a/CD18 heterodimer) in the CD4 T cell immune synapse results in a significant increase in TCR activation (49, 54, 55). TCR activation, actin dynamics and integrin adhesion in the periphery clearly increase further TCR:MHCI interactions, potentiating signaling and synapse lifetime. This may suggest why PI(3,4,5)P3 supported actin dynamics in the distal synapse region is important for CTL killing.

## Diacylglycerol at the Immune Synapse

As already mentioned phosphoinositide changes play a role in directing granules to the synapse. DAG, generated by the initial cleavage of PI(4,5)P2, controls the movement of the centrosome to the synapse membrane through its activation of kinases and motor proteins (56–58). Inositol 1,4,5-trisphosphate (IP3) either binds receptors in the endoplasmic reticulum activating calcium release from intracellular stores or is converted into IP4 and involved in ITK signaling (59). The importance of DAG is clear from studies in which the conversion of PI(4,5)P2 to DAG or un-caging of DAG (independent of calcium signaling) by fluorescence activation are sufficient to polarize the centrosome to the plasma membrane (58). Computer modeling of DAG in membranes suggests that DAG is a mediator of gross biophysical changes within the membrane due to changes in head group spacing and hydrophobic fatty acid packing. These changes in local membrane spacing would allow for improved localization of the DAG dependent protein kinase C (PKC) family members and C1 domain containing proteins (60). DAG plays many other roles at the synapse, supporting the activation of PKC family members, protein kinase D (PKD) and RAS guanyl-releasing protein/RasGRP (57, 61, 62).

Production of DAG at the T cell synapse membrane is important for recruiting PKD. PKCβ, which is a transient resident of the synapse, activates PKD at the synapse after DAG binding (57, 62). There are multiple targets for PKD after activation including transcription factors, the actin cytoskeleton and multiple serine/threonine and tyrosine kinases. This suggests that formation of DAG at the synapse has a significant effect on the post-translational and transcriptional profile of the CTL (63).

Recruitment of the Tec kinase, ITK relies on PI(3,4,5)P3 for its localization and this is enhanced by pleckstrin homology (PH) domain binding of IP4. Inositol 1,4,5-trisphosphate (IP3) 3-kinase B (ItpkB) generates IP4 after PLCγ1 converts PI(4,5)P2 to DAG and IP3. T cells from ItpkB deficient mice were unable to recruit ITK to the immune synapse. T cells recruited less DAG at the synapse and displayed reduced Erk signaling, which may explain why ItpkB null mice have a T cell deficiency (64). This suggests that ItpkB and ITK may act as a signal multiplier, supporting large-scale activation of PLCγ1 at the synapse. This in turn generates more substrate for ItpkB that supports further ITK activation. ITK null CTLs show reduced killing capacity suggesting it does not only affect differentiation of T cell or T cell selection but also regulates effector cell function (65). Mathematical modeling of how IP4 levels play a role in enhancing ITK activation through increased PI(3,4,5)P3/PH domain binding, indicated there is also a negative feedback loop regulating the pathway. As IP4 increases due to continued activation of PLCγ1 and activity of ItpkB, IP4 outcompetes the PI(3,4,5)P3 binding site in the PH domain. This results in dissociation of ITK from the membrane and loss of PLCγ1 activation through ITK activity (66). ITK/ItpkB/IP4/ may therefore regulate the total available DAG created at the membrane during synapse formation independently of other signaling events and ITK through this signaling negative feedback loop may regulate T cell activation and lipid dynamics. Pleckstrin2 is also recruited to the plasma membrane including the synapse of Jurkat cells via the PH domain, co-localizing with actin (67) although the mechanism is not as thoroughly studied as for Itk yet.

## Phosphatidylserine and Phosphatidic Acid at the Synapse

Several other species of phospholipids contribute to membrane specialization in cells including PI(5)P, PI(3)P, PS, PA, and PI(3,5)P2 (Figure 1). Many have specific functions in pathways such as endosome sorting, membrane charge, endocytosis and autophagy. PS is a negatively charged phospholipid; however, it is not significantly depleted from the central synapse region as negative charge decreases (10, 33). It is possible that the influx of positive ions (such as Ca2+ or Mg2+) during TCR signaling may neutralize the charge of these lipid head groups during the charge depletion event, as seen in macrophages where calcium influx limited plasma membrane charge (32, 33, 68). In some CTL, PS appeared to concentrate at the actin-rich distal region of the synapse; F-actin is reported to slow down PS dynamic in the membrane as PS transiently binds with actin-membrane linker proteins (69).

PA, which is the product of DAG phosphorylation by DGK, does not appear to change across the synapse as it forms, suggesting that DAG is not significantly modified during synapse formation (10). This is important, as PA in the plasma membrane has been shown to regulate PIP5K activity (70). As PA does not increase while actin recovers this implies that it is unlikely to be responsible for the recovery of PIP5K, PI(4,5)P2, and actin observed after granule secretion (8).

Several lipids are yet to be visualized in the synapse in T cells as the probes used to investigate their distribution are not specific (71) or express poorly in CTLs. However, it has been shown that phosphatidylinositol 5-phosphate (PI(5)P) can be produced via other routes including the 4'-phosphatase activity of Tmem55a/b on PI(4,5)P2 (Figure 1); myotubularin 3'phosphatases acting on PI(3,5)P2 and inhibition of PIP4K activity or the expression of IpgD (72–74). Interestingly, PI(5)P increases have been shown to lead to increases in cytokine production and Src family kinase signaling in activated T cells (72, 75, 76). Until specific protein sensors become available, information on these lipids is limited.

## Membrane Specialization Across the Primary Cilium and the Immune Synapse

One of the most remarkable findings about the phosphoinositide specialization observed across the synapse is its striking similarity to the phosphoinositide signature across the primary cilium with PI(4,5)P2 and PI(3,4,5)P3 depleted relative to other areas of the plasma membrane in both cilia and the synapse. Primary cilia are specialized sensory organelles that extend from the plasma membrane into the extracellular space that are important for hedgehog and platelet derived growth factor signaling in cells. Primary cilia accumulate receptors in to a specialized region of the membrane concentrating many signaling molecules important for development (77–80). While cells readily produce primary cilia in response to stress or starvation, cells of the haemopoietic lineage do not. This is somewhat surprising as studies in immortalized B and T-cells have demonstrated that these hematopoietic cells do contain the machinery required to form cilia (81).

**Figure 3:**
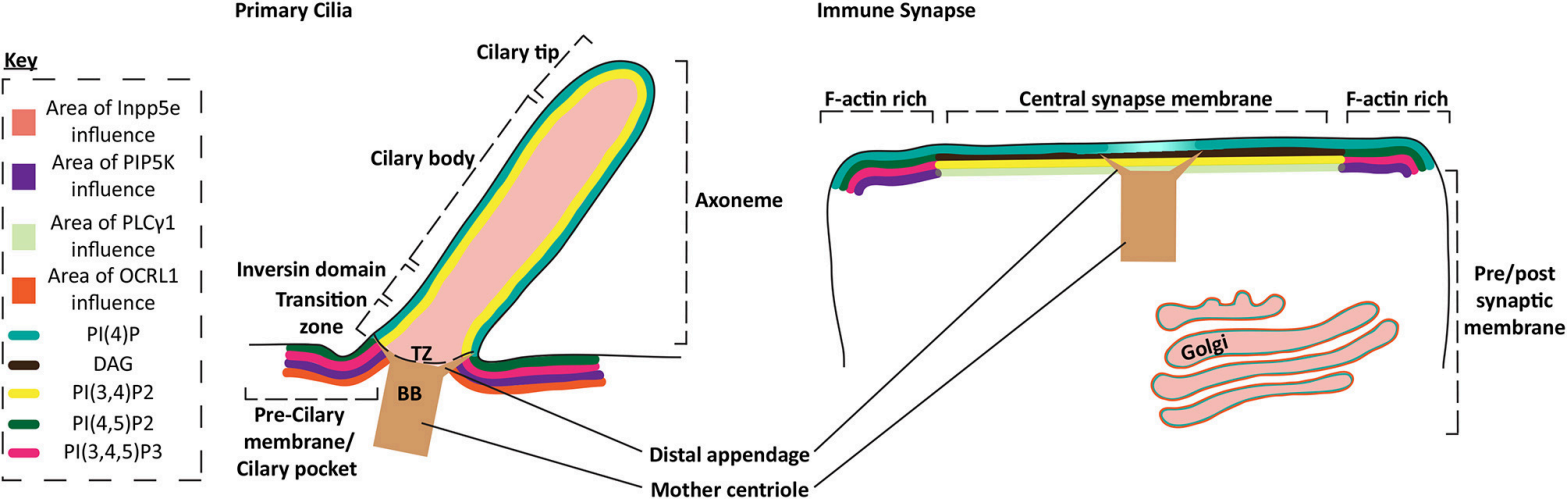
Protein and phospholipid content of primary cilia and the immune synapse. Schematic of the membrane phospholipid content in the primary cilium and the immune synapse. Phospholipid enriched regions of the membrane are represented by the key, while protein areas of influence are also highlighted by colored membranes or regions. Specific membrane regions or ciliary domains are defined through bracketing. PI(3,4)P2 localization in the cilium is inferred from the description of PI(3,4,5)P3 localization and the proposed activity of the Inpp5e in the cilium which converts PI(3,4,5)P3 into PI(3,4)P2, although this could diffuse away or be converted into other metabolites.

Cilia formation is dependent on the activities of Inpp5e and Inositol polyphosphate 5-phosphatase/OCRL1/Inpp5f (5' lipid phosphatases), which convert PI(4,5)P2 and PI(3,4,5)P3 into PI(4)P and PI(3,4)P2, respectively (82–86). This acts to exclude concentrations of PI(4,5)P2 and PI(3,4,5)P3 to the transition zone at the basal body, in favor of PI(4)P and possibly PI(3,4)P2. The localization of PI(3,4)P2 in the cilium is yet to be shown, but must be present if PI(3,4,5)P3 is also converted (Figure 3). Furthermore, Bardet-Biedl Syndrome protein 5, part of a complex thought to transition G-protein coupled receptors into the cilium across the transition zone (87), have PI(3,4)P2 binding PH domains implicating the presence of PI(3,4)P2 in the cilium or as a regulator of protein transport to the cilium (88). Initiation of ciliary signaling and dissolution of cilia results in a shutdown of the lipid phosphatase pathway and production or entry of PI(4,5)P2 into the ciliary membrane. Actin is recruited to cilia after PI(4,5)P2 accumulation (89, 90). After lytic granule delivery, actin is also recovered over the synaptic membrane of CTLs. This suggests that actin is an effective barrier to signaling at the cilia and synapse (7). Interestingly the centrosome uses the interplay between PIP5K and Inpp5e to control the balance of PI(4)P and PI(4,5)P2 in either Golgi associated vesicles or at the plasma membrane. These lipids differentially regulate the recruitment of the distal appendage protein Cep164 or Tau tubulin kinase 2 to mediate ciliation after appropriate signaling occurs (91). In T cells PIP5K and levels of PI(4,5)P2 play a similar role in synapse formation (10). These findings highlight the similarities in membrane specialization in the immune synapse and cilia, supporting the idea that there may be a functional link between the structures.

After granule delivery to the CTL membrane both PI(4,5)P2 and actin are restored across the synapse. Likewise, it appears that actin is only present before formation or after dissolution of cilia (90, 92, 93). This suggests actin may also act as a significant barrier in cilia, also regulated by changes in PI(4,5)P2 and PI(3,4,5)P3 (94) (Figure 3). Interestingly, actin dynamics slowed the flow of receptors transitioning between the cilium membrane and the plasma membrane peripheral to the cilia (93), while actin flow is also thought to play an important role in the coalescence of TCR clusters in T cells (95, 96).

## The Future of Lipids at the Synapse

Phospholipids and their associated signaling molecules transmit information and support the accumulation of protein signaling platforms. They also shape the actin cytoskeleton and thereby sculpt higher order structures in cells. The lipases, kinases, and phosphatases involved in phospholipid metabolism shape the inner leaflet of the membrane without the need for structural proteins that mediate diffusion barriers. Many questions remain, and there are gaps in our knowledge that are not understood from the current literature, or via the use of exogenously expressed, available bio-probes. For example how do TCR complexes that depend on PI(4,5)P2 transition from the periphery, into the cSMAC, where PI(4,5)P2 is depleted? Does depletion of phospholipid head groups result in significant biophysical changes in the membrane? Understanding these mechanisms will provide unique insights as to how regulators of granule delivery could be targeted, perhaps even therapeutically, and may also hold information that transfers to a diverse portfolio of cellular organelles in a diverse range of cells.

## Author Contributions

All authors listed have made a substantial, direct and intellectual contribution to the work, and approved it for publication.

### Conflict of Interest Statement

The authors declare that the research was conducted in the absence of any commercial or financial relationships that could be construed as a potential conflict of interest.

## Abbreviations

MHC I: Major histocompatability complex I
TCR: T cell receptor
CTL: Cytotoxic T lymphocyte
PI(3)P: Phosphatidylinositol 3-phosphate
PI(4)P: Phosphatidylinositol 4-phosphate
PI(5)P: Phosphatidylinositol 5-phosphate
PI(4,5)P2: Phosphatidylinositol 4,5-bisphosphate
PI(3,4,5)P3: Phosphatidylinositol 3,4,5-trisphosphate
PI(3,4)P2: Phosphatidylinositol 3,4-bisphosphate
PI(3,5)P2: Phosphatidylinositol 3,5-bisphosphate
DAG: Diacylglycerol
PS: Phosphatidylserine
PA: Phosphatidic acid
IP3: Inositol 1,4,5 trisphosphate
IP4: Inositol 1,3,4,5 tetrakisphosphate
APC: Antigen presenting cells
LAT: Linker for activation of T cells
PLCγ1: Phospholipase C gamma 1
ItpkB: Inositol 1,4,5-trisphosphate 3-kinase B
PI3K: Phosphatidylinositol 4,5-bisphosphate 3-kinase
PIP4KII: Phosphatidylinositol 5-phosphate 4-kinase
PIP5K: Phosphatidylinositol 4-phosphate 5-kinase
DGK: Diacylglycerol kinase
PTEN: Phosphatidylinositol 3,4,5-trisphosphate 3-phosphatase and dual-specificity protein phosphatase
SHIP1: SH2 domain-containing inositol 5′-phosphatase 1
FERM: 4.1 protein/Ezrin/Radaxin/Moesin
ERM: Ezrin/Moesin/Radaxin
WASp: Wiskott-Aldrich syndrome protein
TIRFM: Total internal reflection microscopy
PKC: Protein kinase C
PKD: Protein kinase D
IFT20: Intraflagella transport 20
ICAM-I: Intercellular adhesion molecule I
TAPP1: Tandem PH domain-containing protein 1
PH domain: Pleckstrin homology domain
Grp1: General receptor of phosphoinositides 1
FYVE: Fab 1/YOTB/Vac 1/EEA1
F-actin: Filamentous actin
Zap70: 70 kDa zeta-chain associated protein
LFA-1: Leukocyte function-associated molecule 1.
